# Comparing outcomes in patients with exsanguinating injuries: an Eastern Association for the Surgery of Trauma (EAST), multicenter, international trial evaluating prioritization of circulation over intubation (CAB over ABC)

**DOI:** 10.1186/s13017-024-00545-8

**Published:** 2024-04-25

**Authors:** Paula Ferrada, Alberto García, Juan Duchesne, Megan Brenner, Chang Liu, Carlos Ordóñez, Carlos Menegozzo, Juan Carlos Salamea, David Feliciano

**Affiliations:** 1 Surgery Service line, Inova Healthcare System, Falls Church, VA USA; 2 Department of Surgery, Trauma and Critical Care, Fundación Valle del Lili, Cali, Colombia; 3 Department of Surgery, Tulane Health System, New Orleans, LA USA; 4 UCLA David Geffen School of Medicine, Los Angeles, CA USA; 5 Division of General Surgery and Trauma, University of Sao Pablo, Sao Pablo, Brazil; 6 Universidad del Azuay, Cuenca, Ecuador; 7 University of Maryland, Shock Trauma Center, Baltimore, MD USA; 8 Division and System Chief, Trauma and Acute Care Surgery, University of Virginia, Inova Healthcare System, 3300 Gallows Road, Falls Church, VA 22042 USA

## Abstract

**Introduction:**

Hemorrhage is a major cause of preventable trauma deaths, and the ABC approach is widely used during the primary survey. We hypothesize that prioritizing circulation over intubation (CAB) can improve outcomes in patients with exsanguinating injuries.

**Methods:**

A prospective observational study involving international trauma centers was conducted. Patients with systolic blood pressure below 90 who were intubated within 30 min of arrival were included. Prioritizing circulation (CAB) was defined as delaying intubation until blood products were started, and/or bleeding control was performed before securing the airway. Demographics, clinical data, and outcomes were recorded.

**Results:**

The study included 278 eligible patients, with 61.5% falling within the “CAB” cohort and 38.5% in the “ABC” cohort. Demographic and disease characteristics, including age, sex, ISS, use of blood products, and other relevant factors, exhibited comparable distributions between the two cohorts. The CAB group had a higher proportion of penetrating injuries and more patients receiving intubation in the operating room. Notably, patients in the CAB group demonstrated higher GCS scores, lower SBP values before intubation but higher after intubation, and a significantly lower incidence of cardiac arrest and post-intubation hypotension. Key outcomes revealed significantly lower 24-hour mortality in the CAB group (11.1% vs. 69.2%), a lower rate of renal failure, and a higher rate of ARDS. Multivariable logistic regression models showed a 91% reduction in the odds of mortality within 24 h and an 89% reduction at 30 days for the CAB cohort compared to the ABC cohort. These findings suggest that prioritizing circulation before intubation is associated with improved outcomes in patients with exsanguinating injuries.

**Conclusion:**

Post-intubation hypotension is observed to be correlated with worse outcomes. The consideration of prioritizing circulation over intubation in patients with exsanguinating injuries, allowing for resuscitation, or bleeding control, appears to be associated with potential improvements in survival. Emphasizing the importance of circulation and resuscitation is crucial, and this approach might offer benefits for various bleeding-related conditions.

## Introduction

Approximately 2 billion people, primarily in rural and marginalized populations within low- and middle-income countries (LMICs), lack access to emergency and essential surgical care [1]. Shockingly, the poorest one-third of the global population receives a mere 3.5% of all surgical procedures, leaving them vulnerable to exsanguination-related fatalities [2, 3]. This threat is not confined to trauma alone, as obstetric, and gastrointestinal emergencies also contribute to hemorrhage-related deaths [1–3]. In the field of trauma, 40% worldwide are related to exsanguination. Hemorrhage stands as the leading cause of preventable death in traumatic scenarios [3–5].

Recent insights have spurred a reevaluation of the primary survey sequence of trauma (ABCs), favoring the prioritization of resuscitation and circulation (CAB) and, when possible, delaying intubation in patients with exsanguinating injuries [6–11]. Prioritizing circulation is a multifaceted approach based on current data suggesting that euvolemic, hemostatic resuscitation strategies using blood products over crystalloid fluids and delaying intubation until either blood products are given or a procedure to stop or slow down bleeding has been performed can improve outcomes in patients with severe traumatic injuries [5, 11, 12]. Intubation in patients who are exsanguinating could worsen hypotension due to vasodilation and decreased venous return secondary to the drugs used for the procedure, as well as the positive pressure ventilation required after intubating a patient [13, 14]. This post-intubation hypotension has been shown to be deleterious in patients with already high morbidity and mortality [13–15]. A conservative management of the airway using other adjuncts could perhaps help the clinicians delay intubation until the hemodynamic state of the patient can tolerate the physiological insult [13–15].

Studies comparing the traditional ABC approach to the CAB model indicate that prioritizing circulation is effective, particularly in trauma patients with hemorrhagic shock [7, 11, 16–20]. This evolving approach underscores the importance of rethinking intubation timing, emphasizing evidence-based practices, rapid response, and collaboration for enhanced patient survival in severe bleeding scenarios [7, 11, 16–21].

We hypothesize that prioritizing circulation over intubation (CAB vs. ABC) in patients with exsanguinating bleeding can improve patient outcomes.

## Methods

This study, approved by the Eastern Association for the Surgery of Trauma (EAST) Multicenter Trials Committee in 2018 and with Institutional Review Board approval, prospectively collected data from six trauma centers: Inova Healthcare System in Falls Church, VA; Fundación Valle del Lili in Cali, Colombia; Tulane Health System in New Orleans, LA; University of California, Los Angeles (UCLA) David Geffen School of Medicine in Los Angeles, CA; University of Sao Paulo in Sao Paulo, Brazil; and Universidad del Azuay in Cuenca, Ecuador. In accordance with the Declaration of Helsinki we did not include vulnerable groups and the study was prospective and observational.

Patients included in the study (from January 2018 to April 2022) were trauma patients with a systolic blood pressure lower than 90 mm Hg, intubated within 30 min of arrival. Recorded data encompassed demographics, mechanism of trauma, injury severity score (ISS), initial Glasgow coma scale (GCS), systolic blood pressure (SBP) at arrival and after intubation, blood received in the first six hours, type of intubation (drug-assisted or not), mortality within the first 24 h, complications (renal failure and acute respiratory distress syndrome [ARDS]), and mortality between the 2nd and 30th day. Prioritizing circulation (CAB) was defined as delaying intubation until blood products were started and/or bleeding control was performed before securing the airway, and this group was compared with patients in whom intubation was prioritized over circulation by intubating before resuscitation or any other maneuver (ABC).

Demographic data and baseline characteristics were summarized according to intubation timing, delayed or not delayed. Categorical variables were presented as amounts and percentages, and continuous variables as median and interquartile range (IQR) after verifying non-normal distribution through the S-K test.

All study endpoints (mortality at 24 h, between the 2nd and 30th day, 30-day mortality, renal failure development, and ARDS occurrence) were compared between delayed and not delayed intubation groups using Fisher’s exact test. Multiple logistic regression models were constructed for significant differences, with statistical significance adjusted according to Bonferroni at a 0.01 level (Curran-Everett).

The diagnosis of final models involved scrutiny for specification errors, influential observations, and multicollinearity (Hosmer, Zhang). The discriminative ability was assessed by the area under the receiver operating characteristic curve (AUROC), and goodness-of-fit (GOF) was evaluated by the Hosmer-Lemeshow test (Hosmer). Results are reported as odds ratios (OR) with their 95% confidence interval.

All statistical analyses were conducted in Stata 15.1 (StataCorp, College Station, TX).

## Results

The analysis encompassed a total of 278 eligible patients, with 171 individuals (61.5%) falling within the CAB cohort and 107 patients (38.5%) in ABC cohort. In terms of geographical distribution, 155 patients were attended to in the United States (55.8%), while 123 patients received care outside of the USA.

Demographic and disease characteristics of all patients as well as those within each cohort are summarized in Table 1. Crucially, variables such as age, sex, ISS, use of blood products, drug-assisted intubation, and resuscitative thoracotomy exhibited comparable distributions between the two cohorts.

**Table 1:** Summary and comparison of demographic and disease characteristics

Characteristics	CAB	ABC	All
Patients, n	171	107	278
Age (Years), Median [Q1, Q3]	46 [36, 61]	50 [33, 61.5]	47 [35, 61]
Sex			
Male, n (%)	150 (87.72)	94 (87.85)	244 (87.77)
Female, n (%)	21 (12.28)	13 (12.15)	34 (12.23)
ISS, Median [Q1, Q3]	43 [29, 57]	42 [30, 55]	42 [29, 56]
Mechanism of Injury			
Penetrating, n (%)	48 (28.07)	17 (15.89)	65 (23.38)
Blunt, n (%), n (%)	123 (71.93)	90 (84.11)	213 (76.62)
Intubation in OR, n (%)	67 (39.18)	0/107 (0)	67/278 (24.1)
Blood Products Before Intubation, n (%)	129 (75.44)	65 (60.75)	194 (69.78)
Blood Products, Median [Q1, Q3]	6 [4, 8]	6 [4, 9]	6 [4, 9]
Drug-Assisted Intubation, n (%)	113 (66.08)	61 (57.01)	174 (62.59)
REBOA, n (%)	9 (5.26)	0 (0)	9 (3.24)
RT, n (%)	7 (4.1)	3 (2.8)	10 (3.6)
GCS Median [Q1, Q3]	8 [6, 11]	7 [5, 9.5]	8 [5.25, 11]
SBP Before Intubation Median [Q1, Q3]	71 [63, 81.5]	76 [66.5, 81.5]	73 [64, 81.75]
SBP After Intubation Median [Q1, Q3]	67 [58, 73]	57 [50, 67]	63.5 [54, 71]
Arrest with CPR, n (%)	17 (9.91)	79 (73.83)	96 (34.5)

Q1, Quartile 1 Q3, Quartile 3 OR, Operating room REBOA, Resuscitative Endovascular Balloon Occlusion of the Aorta RT, Resuscitative Thoracotomy GCS Glasgow Coma Scale SBP, Systolic Blood Pressure CPR, Cardiopulmonary Resuscitation

A higher proportion of patients in the CAB group (28.1%) presented with penetrating injuries compared to the ABC cohort (15.9%). Moreover, 39.2% of patients in the CAB group received intubation in the operating room, in contrast to none in the ABC group. Patients in the CAB group that were not intubated in the operating room, where intubated in the emergency room, however after receiving resuscitation or maneuvers for bleeding control.

A small subset of patients in the CAB group (5.3%) underwent resuscitative endovascular balloon occlusion of the aorta (REBOA), whereas none in the ABC group did. The GCS score was significantly higher in the CAB group (median 8, IQR: 6–11) compared to the ABC group (7, IQR: 5-9.5).

In terms of blood pressure, the CAB group exhibited significantly lower SBP values before intubation (71, IQR: 63-81.5) compared to the ABC group (76, IQR: 66.5–81.5). However, after intubation, SBP in the CAB group was significantly higher (64.8, IQR: 58–73) compared to the ABC group (57, IQR: 50–67). Moreover, the percentage of patients experiencing cardiac arrest requiring cardiopulmonary resuscitation (CPR) was notably lower in the CAB group (10.1%) compared to the ABC group (74.5%). Patients in the CAB group also demonstrated a reduced incidence of post-intubation hypotension (25.2% vs. 51.4%, *p* < 0.001).

Table 2 provides a summary and comparison of the key outcomes of interest. The 24-hour mortality rate was significantly lower in the CAB group, with only 11.1% of patients succumbing within this timeframe, in stark contrast to the ABC group where 69.2% of patients met the unfortunate outcome (*P* < 0.001). Additionally, the CAB group exhibited a lower rate of renal failure (14.6% vs. 22.4%) and a higher rate of ARDS (4.7% vs. 0.9%), although the latter two findings approached statistical significance (*P* = 0.107 and *P* = 0.160, respectively). There was no statistically significant difference in the late mortality rates between the two study groups (mortality between the 2nd and 30th days). Total 30-day mortality was significantly higher in the ABC group (72.0% vs. 17.5%, *p* < 0.001).

**Table 2:** Summary and comparison of outcomes

Characteristics	CAB	ABC	All	*P*-value
24-hour Mortality, n (%)	19 (11.11)	74 (69.16)	93 (33.45)	< 0.001*
Mortality after 24-hour, n (%)	11/152 (7.24)	3/33 (9.1)	14/185 (7.6)	0.718*
30-day Mortality, n (%)	30 (17.5)	77 (72.0)	107 (38.5)	< 0.001*
Renal Failure, n (%)	25 (14.62)	24 (22.43)	49 (17.63)	0.107*
ARDS, n (%)	8 (4.68)	1 (0.93)	9 (3.24)	0.16*

ARDS, Acute respiratory Distress Syndrome *Fisher’s Exact Test

The results from multivariable logistic regression models for 24-hour and 30-day mortality are presented in Tables 3 and 4.

**Table 3:** Results of logistic regression for 24-hour mortality

	Univariable			Stepwise Multivariable		
	Unadjusted OR	95% CI	*P* Value	Adjusted OR	95% CI	*P* Value
CAB	0.06	0.03–0.1	< 0.001	0.09	0.04–0.18	< 0.001
Age	1.0	0.99–1.02	1.0			
Sex (Female vs. Male)	1.68	0.81–3.48	0.163	2.28	0.88–5.87	0.09
Mechanism of Injury (Blunt vs. Penetrating)	2.40	1.23–4.69	0.01			
ISS	0.99	0.98–1.0	0.27			
Blood within 6 h	1.0	0.92–1.09	0.95			
Blood Products Before Intubation	0.69	0.41–1.18	0.18			
Drug Assisted Intubation	0.44	0.26–0.73	0.001	0.43	0.22–0.83	0.012
RT	0.85	0.21–3.36	0.81			
GCS	0.96	0.89 − 0.23	0.27			
SBP, mm Hg	1.02	0.99–1.04	0.11			
SBP after Intubation, mm Hg	0.97	0.95–0.99	0.003			
Arrest with CPR	9.48	5.33–16.89	< 0.001	2.51	1.18–5.32	0.02

OR, Odds Ratio 95% CI, 95% Confidence Interval ISS, Injury Severity Score RT, Resuscitative Thoracotomy GCS, Glasgow Coma Scale

SBP, Systolic Blood Pressure Hg, Mercury CPR, Cardio-Pulmonary Resuscitation

**Table 4:** Results of logistic regression for 30-day mortality

	Univariable			Stepwise Multivariable		
	Unadjusted OR	95% CI	*P* Value	Adjusted OR	95% CI	*P* Value
CAB	0.08	0.05–0.15	< 0.001	0.11	0.05–0.23	< 0.001
Age	1.00	0.99–1.01	0.74			
Sex (Female vs. Male)	1.49	0.73–3.08	0.28			
Mechanism of Injury (Blunt vs. Penetrating)	2.07	1.12–3.83	0.021			
ISS	0.99	0.98–1.01	0.94			
Blood within 6 h	0.99	0.92–1.07	0.84			
Blood Products Before Intubation	0.77	0.46–1.30	0.33			
Drug Assisted Intubation	0.56	0.34–0.92	0.023			
RT	3.92	0.99–15.05	0.051	20.22	2.87–142.6	0.003
GCS	0.95	0.89–1.02	0.182			
SBP, mm Hg	1.02	1.00–1.04	0.056	1.02	0.99–1.05	0.062
SBP after Intubation, mm Hg	0.98	0.95–0.99	0.019			
Arrest with CPR	6.84	3.95–11.85	< 0.001	1.89	0.91–3.95	0.21

OR, Odds Ratio 95% CI, 95% Confidence Interval ISS, Injury Severity Score RT, Resuscitative Thoracotomy GCS, Glasgow Coma Scale

SBP, Systolic Blood Pressure Hg, Mercury CPR, Cardio-Pulmonary Resuscitation

In this regard, the CAB cohort demonstrated a 91% reduction in the odds of mortality within 24 h (aOR: 0.09; 95% CI: 0.04–0.18; *P* < 0.001) and an 89% reduction in the odds of mortality at 30 days (aOR: 0.11; 95% CI: 0.05–0.23: *P* < 0.001) compared to the ABC cohort.

Remarkably, the MLR models exhibited a good discriminative ability: AUROC of 0.86 (95% CI 0.81–0.90) and 0.83 (95% CI 0.78–0.88) for the 24-hour and 30-day models respectively, and a good GOF with a Hosmer (Chi2 = 0.209 and 0.770) for the 24-hour and 30-day models respectively.

## Discussion

This international prospective study suggests that there may be potential benefits in prioritizing circulation over intubation in hypotensive trauma patients with exsanguinating injuries. It is acknowledged that other studies have indicated the advantages of delaying intubation until the patient is in the operating room, particularly for those with exsanguinating injuries [6].

The results demonstrate that a majority of patients experienced post-intubation hypotension (PIH), consistent with findings in previous studies associating PIH with worse outcomes in various patient populations, including trauma [15]. Our data suggests that patients undergoing the CAB approach experienced a smaller drop in systolic blood pressure (SBP) after intubation compared to the ABC group. Patients who have lost intravascular volume due to bleeding rely on compensatory mechanisms to maintain cardiac output and adequate organ perfusion. Endotracheal intubation, with induction drugs, neuromuscular blockade, and positive-pressure ventilation, can potentially disrupt these compensatory mechanisms. Intubating patients who have not been adequately resuscitated and are still bleeding may exacerbate their condition, leading to increased blood transfusions, delays in surgical hemorrhage control, and a higher likelihood of subsequent organ dysfunction.

While our study findings support the idea of delaying intubation, when possible, it is essential to recognize the limitations inherent in our study design and the complexity of patient care. The observed deterioration is secondary to a physiological response, highlighting its relevance for patients with traumatic injuries and potentially extending to those with bleeding from other sources, such as obstetric complications, vascular, or gastrointestinal pathologies.

The results underscore the significance of prioritizing circulation and perfusion during the initial management of patients with exsanguinating injuries, suggesting a potential influence on outcomes. This study contributes to existing research by presenting evidence that delaying intubation in favor of focusing on resuscitation and swift hemorrhage control could potentially impact survival.

Effective airway management in trauma patients facing severe bleeding and hypovolemic shock is crucial for their survival. While immediate airway attention is pivotal in the initial assessment, strategies exist to potentially delay intubation to prioritize resuscitation and bleeding control. Basic airway techniques, high-flow oxygen administration, and other adjuncts can maintain oxygenation and ventilation, allowing time to address bleeding and administer blood products. Urgent airway intervention becomes imperative in specific scenarios, necessitating careful drug-assisted intubation to avert shock escalation.

The proposed CAB approach extends beyond delaying airway intervention to encompass a multifaceted strategy, including early blood product transfusion, minimizing crystalloids, and re-evaluating the prioritization of securing the airway. It suggests that interventions such as providing oxygen, removing upper airway obstructions, and supporting oxygenation and ventilation should be considered as the first line of treatment in patients with exsanguinating injuries, rather than immediate intubation. In cases of severe bleeding, adopting a circulation-airway-breathing (CAB) approach is suggested, prioritizing the gravest concern first. Whenever feasible, timely resuscitation with blood products is emphasized for its potential advantages in improving survival rates and reducing blood product needs in trauma patients.

### Limitations

We acknowledge that our study has certain limitations that should be taken into consideration. It is important to note that our research was non-randomized, and this design introduces the possibility that the observed outcomes in immediate intubation patients could be influenced by variations in their initial health status upon admission. It is crucial to emphasize that our work was conducted as an observational trial rather than a randomized controlled trial, which means that our findings should be interpreted within this context. We recognize that, similar to the CAB group, a substantial proportion (60%) of patients in the ABC group also received blood before intubation. This variable adds complexity to the interpretation of our results, and we acknowledge the challenge of disentangling the effects of intubation timing from the influence of pre-intubation interventions, particularly in a non-randomized study design. Furthermore, in clinical practice blood transfusion can be started simultaneously to intubation, it does not need to delay this procedure.

The GCS score was significantly higher in the CAB group (median 8, IQR: 6–11) compared to the ABC group (7, IQR: 5-9.5), Also a higher proportion of patients in the CAB group (28.1%) presented with penetrating injuries compared to the ABC cohort (15.9%). These data suggest that the CAB approach it is likely to be more useful in patients with penetrating injuries and without traumatic brain injury.

Given these limitations, it is not feasible to establish a causal relationship between intubation timing and outcomes definitively. These considerations underscore the need for further investigation and caution in drawing causative conclusions based on the observed associations.

## Conclusions

Conducted as an observational trial, our findings should be interpreted within this context, recognizing the challenge of disentangling the effects of intubation timing from pre-intubation interventions. We acknowledge the non-randomized nature of our research, which introduces the possibility of outcome variations influenced by patients’ initial health status upon admission. Post-intubation hypotension correlates with worse outcomes. Prioritizing circulation over intubation in exsanguinating injuries appears to be associated with improved survival; however, more research in this area is necessary to change the current paradigm. We need to discern which patients benefit more from this approach; in our data, there were more patients with penetrating mechanisms in the CAB group. Emphasizing circulation and resuscitation may offer benefits in bleeding-related conditions, underscoring the need for cautious interpretation and continued research in this area.

## Data Availability

No datasets were generated or analysed during the current study.
